# Baropodometric evaluation of foot load distribution during gait in the group of professionally active nurses

**DOI:** 10.1002/1348-9585.12102

**Published:** 2019-12-14

**Authors:** Anna Kołcz, Natalia Główka, Mateusz Kowal, Małgorzata Paprocka‐Borowicz

**Affiliations:** ^1^ Laboratory of Ergonomics and Biomedical Monitoring Wroclaw Medical University Wroclaw Poland; ^2^ Department of Physiotherapy Wroclaw Medical University Wroclaw Poland

**Keywords:** baropodometric gait analysis, musculoskeletal overload, nordic musculoskeletal questionnaire, nurses, pain syndromes, workplace ergonomy

## Abstract

**Objectives:**

Nurses are extremely exposed to musculoskeletal overloads. Prolonged standing postural balance distributions, functional deficits and pain may affect the symmetry of the load on the feet. The study aimed to assess the distribution of foot load during gait among nurses.

**Methods:**

The sample of this prospective and observational study consisted of 37 female nurses with mean age of 39 years. The Nordic Musculoskeletal Questionnaire (NMQ) was used to evaluate musculoskeletal disturbances and baropodometric gait analysis (BGA) was performed to register distribution of foot load during gait.

**Results:**

We showed that 68% of nurses declare that they know the principles of workplace ergonomics, but only 14% comply with them. NMQ results indicate that as many as 73% of the respondents feel ailments in the "lower back" area. An asymmetry was observed in the load of IV‐V of the metatarsal head between the left and right foot (*P* = .000) and in the load of the left and right lateral part of the heel (*P* = .028) in the BGA test. Correlations between ailments occurring in the neck area and loading of the lateral arch of the right foot (*P* = .032) were found. Moreover, the load in this area correlated positively with the occurrence of "lower back" pain (*P* = .045).

**Conclusions:**

Nurses have asymmetric distribution of foot load during gait, which results in a discrepancy between the loads on the three main support points of the foot and which may affect nurses’ work productivity.

## 1 INTRODUCTION

Healthcare workers often declare poor knowledge and non‐application of ergonomic principles.^1^ The most common reasons are lack of knowledge related to the need to apply ergonomic principles at work, haste or carelessness. Nurses often work with lifting and carrying loads that exceed the body's adaptability.^2^ It is likely that choosing more precise content related to traffic ergonomics and safe behavior during the Occupational Health and Safety (OSH) Training, or increasing the training offer in this area, could raise workers' awareness of occupational safety.^3^, ^4^ The most common mistakes are incorrect body position during professional activities, bad movement habits, non‐use of auxiliary equipment and manual lifting and improper handling of too much weight.^5^

Pain in the structures of the musculoskeletal system is caused mainly by overload mechanisms, which occur very often during work and everyday life activities.^6^ There are far fewer cases of sudden injury, which is the direct cause of pain. Working in a standing position affects the occurrence of overloads of shoulder band muscles and overloads of cervical spine structures. Bend work is a very stressful position for the lumbar region of the spine. If in this position, heavy objects are additionally lifted or carried, the overloads within the structures of the lumbar region of the spine are close to the maximum threshold of tissue strength.^7^

The most common positions taken up by nurses during work are combined positions consisting of several plane components. This alignment of spinal structures leads to their overload and results in disorders of symmetry of skeletal muscle tensions. As a result, pain and dysfunctions of the musculoskeletal system occur.^8^ The majority of active nurses declare that they experience pain in different parts of the spine, of different intensity.^9^ Nurses, due to the physical nature of work and exposure to many harmful factors, are an occupational group particularly exposed to the occurrence of dysfunctions of the movement system.^10^, ^11^, ^12^

There are several studies evaluating body mechanics and spinal kinematics, especially foot architecture and gait parameters, in relation to low back pain regarding occupational health among nurses. Rowe and White^13^ in their biomechanics study, performed three‐dimensional assessment of spinal kinematics during gait to asses movement ranges of the lumbar spine during gait among nurses with musculoskeletal low back pain. It was observed that minimum rather than maximum flexion of the lumbar spine occurred at initial contact and that flexion increased early in the single‐support phase of the gait cycle. Kitagawa et al^14^ studied the link between foot position and load of the L4‐L5 spinal segments as lower back load during supporting standing‐up via musculoskeletal simulation in the group of caregivers. It was shown that as the anteroposterior distance and lateral widths between both feet increased, the average value of compression of the L4‐L5 segments during movements decreased. It confirms that the foot position may reduce the lumbar load and prevent low back pain. Our previous report assessing lower limb load using the baropodometric platform and the body composition with electrical bioimpedance in nurses showed that the forefoot overload was more frequent, the ground peak pressure point was higher and the centre of gravity was shifted. Moreover, the mean visceral fat index in the study group was significantly higher than in controls.^15^

Despite well‐defined legal acts, a large percentage of nurses carry a more substantial burden during on‐call time than that specified in Polish law for women. Approximately 25% of nurses declare that they always lift weights ergonomically, bending lower limbs and keeping the spine straight.^5^ Adoption of forced, asymmetrical body postures and a long‐standing position during work can affect the improper use of the feet through the asymmetry of the distribution of the load on the feet.^16^, ^17^

Functional efficiency of the foot is determined by its musculoskeletal efficiency, bone structure and the loads it undergoes during everyday activity. The method of loading and setting of the foot is often the result of biomechanical relationships concerning other parts of the movement system.^18^, ^19^ For example, structural changes in the spine may cause asymmetry of foot loads, weakening of their muscle, ankle stabilization and gait asymmetry.^20^ That is why taking care of proper shaping and functioning, by controlling the symmetry of load on the feet, preventing their deformations and a thorough analysis of shaping and load on the feet may support the feeling of comfort and safety in the work of the professional nurses' group.

The study aimed to assess the distribution of foot load during gait among active nurses.

## 2 SUBJECTS AND METHODS

### 2.1 Design and settings

This prospective and observational study was conducted at the Department of Physiotherapy at the Wroclaw Medical University in Poland from September 2017 to December 2017. The Clinic has been adapted to the study performed by separating the place to perform measurements, to fill in questionnaires and to rest before the study. The STROBE guidelines (Strengthening the Reporting of Observational Studies in Epidemiology) were followed.^21^

### 2.2 Qualification criteria

Inclusion criteria were: (a) active nurse status; (b) no diagnosed chronic systemic diseases; (c) no diagnosed metabolic disorders; (d) no swelling due to venous or lymphatic insufficiency; and (e) voluntary written consent to take part in the study. In turn, the exclusion criteria comprised: (a) no professional activity in the nursing position; (b) presence of contraindications for measurement and (c) no consent to take part in the study.

### 2.3 Study participants

The study sample consisted of 37 women studying nursing in part‐time mode at the Faculty of Health Sciences of the Wroclaw Medical University in Poland. The subjects were familiarized with the test procedure and agreed to carry it out.

### 2.4 Measurement tools

The test procedure consisted of four parts. An interview was conducted to obtain information on age, seniority, knowledge, and use of ergonomics at work, working time per week, working time spent standing during the day, the type of footwear used in the course of work and how to deal with pain. The height and body weight were measured, and the BMI calculated according to the formula BMI = kg/m^2^. A standardized Nordic Musculoskeletal Questionnaire (NMQ) for the analysis of musculoskeletal symptoms was used.^22^ An objective baropodometric gait analysis (BGA) was also performed to evaluate the reaction of ground forces using a posturographic platform.^23^

#### 2.4.1 Nordic musculoskeletal questionnaire

Standardized NMQ was used to evaluate the musculoskeletal ailments. The results obtained in the first part of the questionnaire on feelings of pain, stinging, discomfort or numbness (hereinafter referred to as pain) in the last 12 months in specific areas of the body (neck, shoulders, upper back, elbows, wrists/arms, lower back, hips/thighs, knees, ankles/foots) were used for the analysis.^24^

#### 2.4.2 Baropodometric gait analysis

An objective analysis of BGA parameters was carried out using the FreeMed Professional platform (Sensor Medica, Rome, Italy). It allows for the analysis of the distribution of foot pressure on the ground. The platform consists of three elements: a mat equipped with resistance sensors and two smaller passive mats, located at the beginning and the end of the path. Technical specification includes: alimentation: 15Vcc; current consumption: 300 mA; resolution XY: 2.5 dpi; resolution Z: 8 bit; acquisition frequency 5‐400Hz selectable; dimension: 1840 × 740 mm; thickness: 8 mm; weight: 16 kg; length: 180 cm; width: 50 cm; type of sensor: resistive conductive rubber contacts gold; type of scanning: matrix scan; calibration: 10 bit auto; suitable temperature working 0°C‐55°C; maximum pressure 150 N/cm^2^; and Product Certification: CE. The platform is connected to a computer, where the transmitted data is saved and analyzed in FreeStep software (Sensor Medica, Rome, Italy).

**Figure 1:**
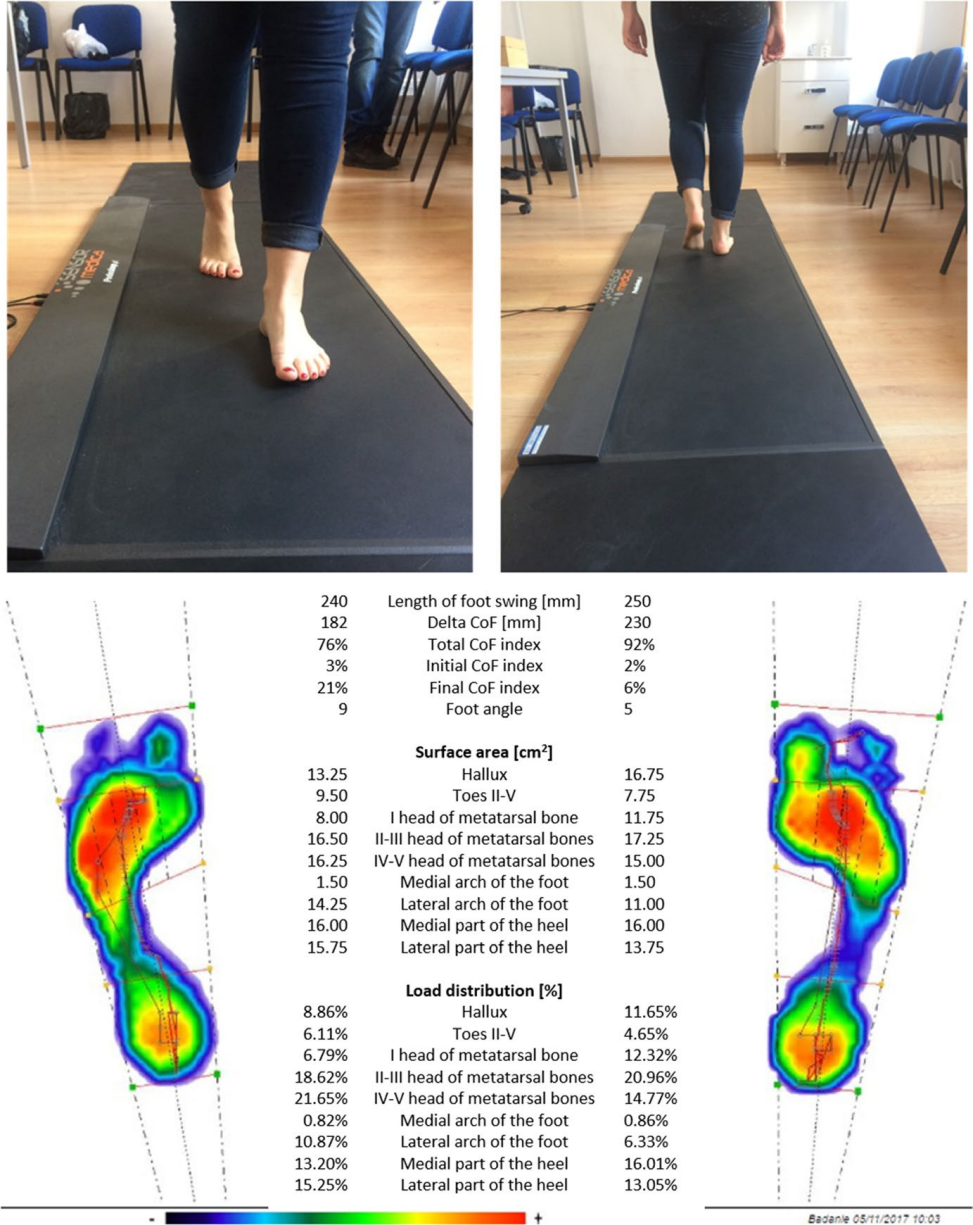
Example of the test procedure and the result of the baropodometric test

The test procedure was to walk on the surface of the platform without shoes. The pace of walking was not imposed. The test procedure started with the passage of two preliminary platform lengths, without recording the BGA measurement data, in order to achieve the greatest possible freedom of movement during the walk. Then the recording was started, and the persons surveyed were asked to make a six‐fold walk along the entire length of the platform path^25^, ^26^ (Figure 1).

During the walk, the sensors collect data about foot pressure on the ground, which is then transmitted and saved in the FreeStep software (Sensor Medica, Rome, Italy). The sampling frequency was set at 400 Hz (400 samples/s) according to the manufacturer's instructions. During the test procedure, the following results were obtained: (a) average maximum loads [gr/cm^2^], (b) average loads [gr/cm^2^], (c) average foot area [cm^2^] and (d) right and left foot load distribution [%]. The foot is divided into nine areas corresponding to the anatomical parts of the sole foot: (a) hallux; (b) toes 2‐5; (c) I head of metatarsal bones; (d) II‐III head of metatarsal bones; (e) IV‐V head of metatarsal bones; (f) medial arch of the foot; (g) lateral arch of the foot; (h) medial part of the heel; and (i) lateral part of the heel.

As part of the further statistical analysis, the whole study group was divided into two age groups, ie, younger people ≤40 years of age (n = 16, 43%) and older people ≥41 (n = 21, 57%). A comparative analysis of the load on sole individual areas of the left and right feet in both age groups was performed. Statistically significant differences were observed in the load on IV‐V of the metatarsal bone head and the lateral part of the heel.

### 2.5 Ethical considerations

The study was approved by the independent Bioethics Committee at the Wroclaw Medical University in Poland (no. KB‐148/2018) and was conducted according to the Declaration of Helsinki and Good Clinical Practice guidelines. All participants gave their informed consent to participate in this study.

### 2.6 Statistical analyses

The obtained data was encoded and transferred to MS Office Excel 2017 and then subjected to statistical analysis using Statistica version 13.3 (TIBCO Software Inc, United States). Descriptive statistics including mean (M), standard deviation (SD), median (Me), minimum (Min) and maximum (max) for participants’ characteristics and results for load of the foot in BGA were shown. The collected data for the load of metatarsal bones IV‐V and load of the lateral part of the heel, as well as the load of the head of metatarsal bone I, toes II‐V, the medial and lateral arch of the foot were statistically were analyzed using the Wilcoxon test. Also, r‐Pearson correlation coefficient was calculated between selected BGA variables and NMQ results. Statistical significance was set at *P* < .05 and strength of correlation was assumed for |*r* < .2| as significant relationship. On the basis of the analysed parameters, the estimated sample size has been obtained equal to 35 nurses to collect data based on NMQ and BGA measurements with relevant difference.

## 3 RESULTS

### 3.1 Participants’ characteristic

The average age of the respondents was 39 years. The average body height was 166 cm, and the average body weight was 67 kg. The average value of BMI in the study group was 24 kg/m^2^. More than a half, ie, 54% (n = 20) of the nurses surveyed had work experience greater than 20 years, 24% (n = 9) were nurses with work experience of less than 2 years. Full characteristics of the study group are described in Table 1.

**Table 1:** Characteristics of the study group

Character	M	Me	Min	Max	SD
Age [y]	39	43	22	55	11.4
Height [cm]	166	166	156	180	0.06
Weight [kg]	67	64	50	104	11.6
BMI	24	23	18	36	3.8

Abbreviations: BMI, body mass index; M, mean; Max, maximum; Me, median; Min, minimum; SD, standard deviation.

Over 40% (n = 15) of the nurses surveyed declared that they spend 40 to 50 hours a week at work; the same percentage spend 30‐40 hours a week. As many as 41% (n = 15) of the respondents declared that they spend more than 8 hours a day standing position, the other as much in this position from 5 to 8 hours a day. As many as 49% (n = 18) of respondents declared that during work they wear flaps, which are not shoes ensuring proper stabilization of the foot during the walk, do not support the natural work of the foot during the walk, basically make it impossible to start the step from the heel, and do not ensure proper cushioning. Only 43% (n = 16) use profiled medical footwear, ensuring appropriate conditions for the proper functioning of the foot.

It was observed that 68% (n = 25) of respondents declare that they know the principles of ergonomics. However, only 14% (n = 5) admitted that they always apply ergonomic principles in the course of their work, 62% (n = 23) sometimes apply these principles, and 24% (n = 9) of respondents consciously do not apply ergonomic principles in the course of their work.

### 3.2 Musculoskeletal condition

On the basis of the results obtained from the NMQ standardized questionnaire concerning the diagnosis of painful areas, such as tingling, discomfort or numbness, occurring in the last 12 months in specific areas of the body, it appears that as many as 73% (n = 27) of the respondents have ailments related to the "lower back" area, ie the lumbar spine, 62% (n = 23) of the nurses surveyed feel these ailments in the neck area, 57% (n = 21) of the respondents suffering from "upper back", ie cervical and shoulder area, 38% (n = 14) indicated knee ailments, 22% (n = 8) suffer from hips and thighs, and 16% (n = 6) suffer from ankle and foot ailments.

### 3.3 Foot load distribution

During the BGA, data were obtained on the average foot surface area [cm^2^], average maximum foot loads [gr/cm^2^] and the mean value of average foot loads [gr/cm^2^]. Average results for the whole study group presented in Table 2 express the above‐mentioned data for the right and left foot. Minimal differences between the tested parameters of both feet can be observed.

**Table 2:** Average area, average maximum loads and average of mean loads for left and right foot

Parametr	M	Me	Min	Max	SD
Parameter	59	57	44	81	7.9
Average area L [cm^2^]	60	59	43	82	7.8
Average area P [cm^2^]	1423	1416	1127	1946	178.7
Average maximum loads L [gr/cm^2^]	1419	1446	1054	1935	183.8
Average maximum loads P [gr/cm^2^]	778	776	642	987	80.8
Average mean loads L [gr/cm^2^]	775	774	606	968	83.6

Abbreviations: L, left; M, mean; Max, maximum; Me, median; Min, minimum; R, right; SD, standard deviation.

**Figure 2:**
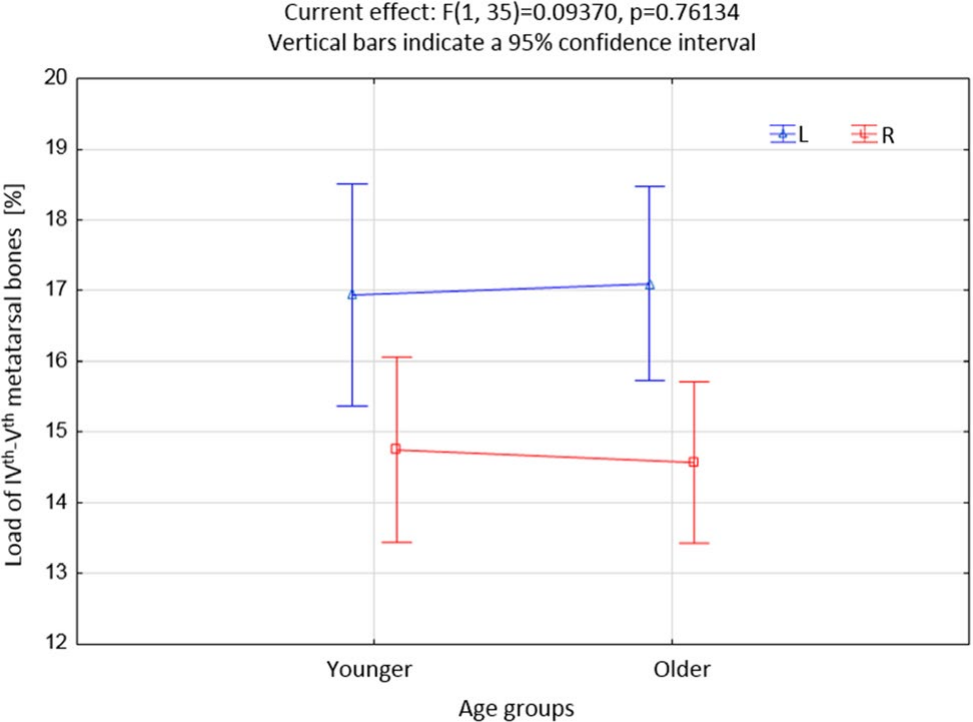
Load of metatarsal bones IV‐V in both age groups [%]

**Figure 3:**
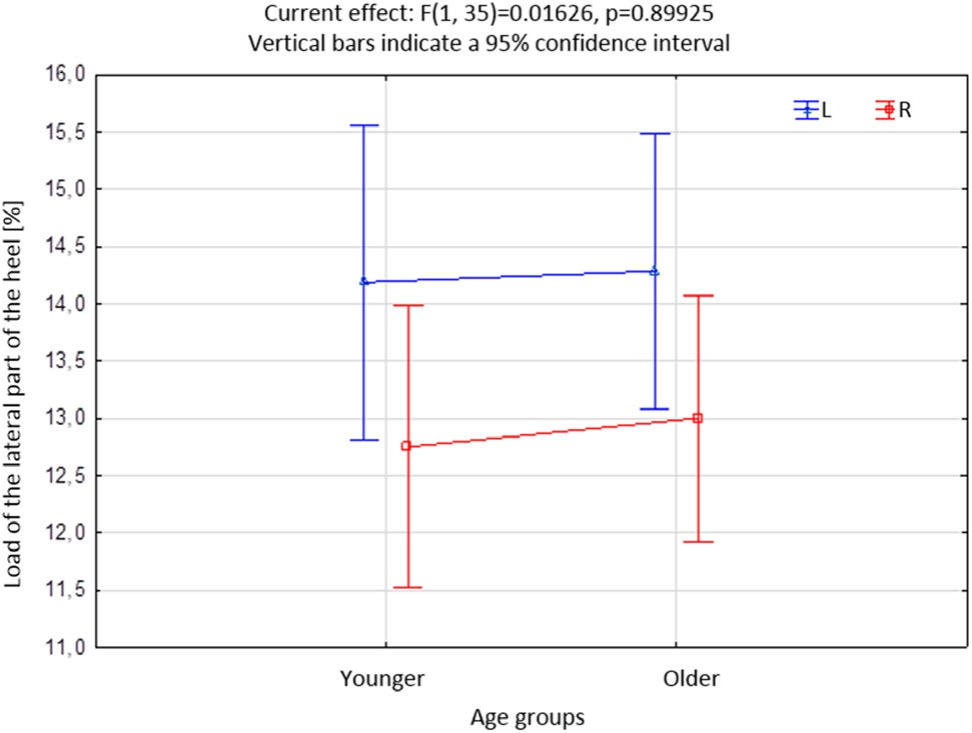
Load of the lateral part of the heel in both age groups [%]

Figure 2 shows the asymmetry in the load of IV‐V of the metatarsal bones between the left and right foot. IV‐V the head of the metatarsal bone in the left foot is always (regardless of the age group) loaded more than the same area of the right foot (*P* = .000). Figure 3 shows the occurrence of asymmetry in the load on the left and right side of the heel. As before, the area in the left foot is loaded to a greater extent than in the right foot (*P* = .0283), regardless of the age of the subjects.

**Figure 4:**
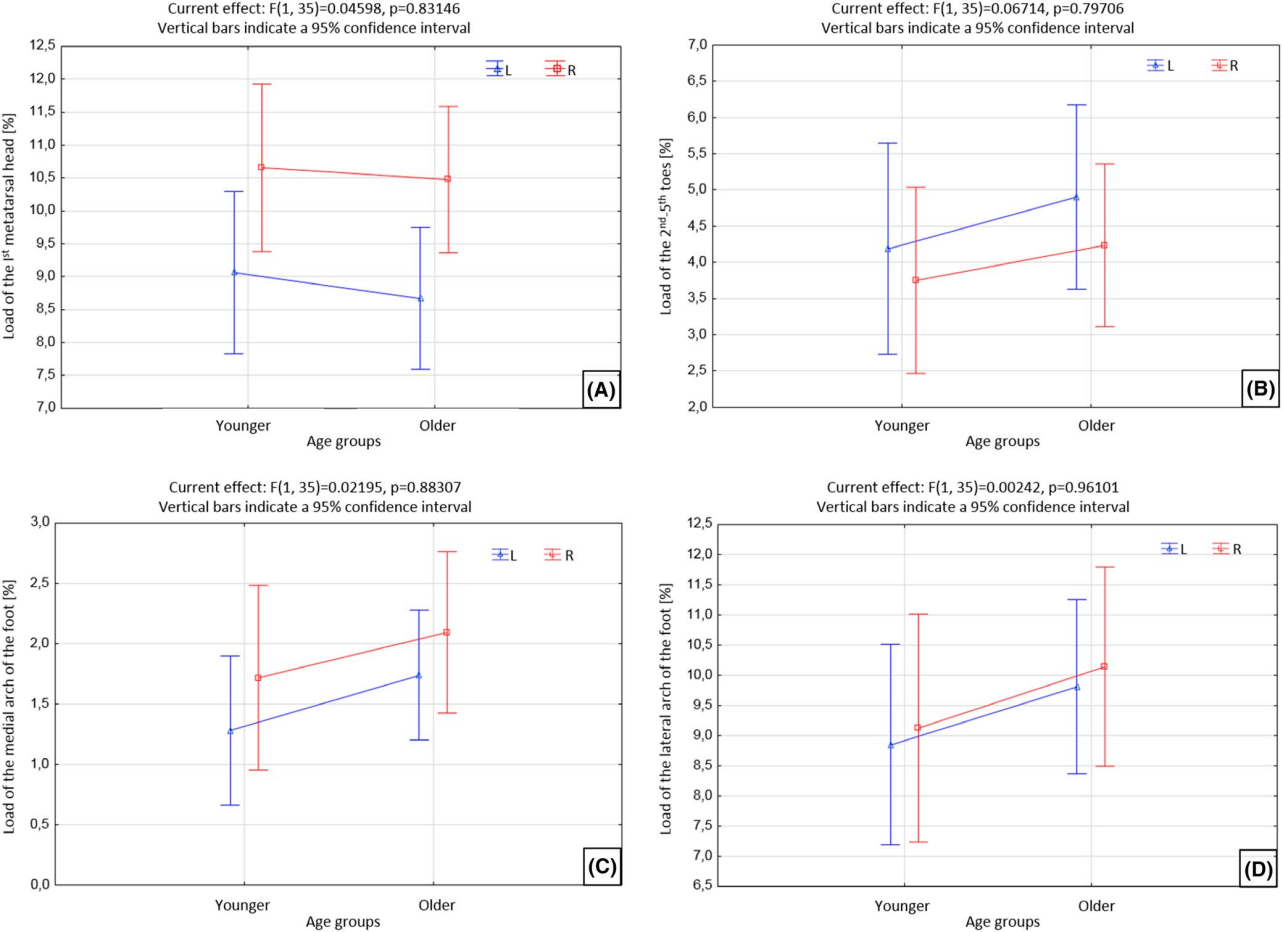
Load of the head of metatarsal bone I (A), toes II‐V (B) of the medial arch of the foot (C) and lateral arch of the foot (D) in both age groups [%]

There were no statistically significant differences between the load on the metatarsal region of the first head of metatarsal bones in the left and right foot. However, there is a tendency to increase the load on this area in the right foot in both age groups (Figure 4A). There were no statistically significant differences in the burden on individual foot areas in both age groups. On the other hand, a tendency can be observed in the group of older nurses to strain the finger areas 2‐5 (Figure 4B) and the medial arch (Figure 4C) and lateral foot (Figure 4D) to a greater extent than in the group of younger nurses. Perhaps it is related to footwear worn at work, ie, flaps that limit the physiological work of the foot.

### 3.4 Correlation between variables

Pain and discomfort in the last 12 months in the body areas described in the NMQ and the load areas on the sole side of the feet were also statistically analyzed. There was a statistically significant relationship between ailments occurring in the neck area and the load on the lateral arch of the right foot (*P* = .032). It was observed that the loaded area of the lateral arch of the right foot correlated positively with the occurrence of pain in the "lower back." (*P* = .045). A statistically significant relationship was found between knee pain and load on the lateral arch of the left foot (*P* = .020) and right foot (*P* = .044).

Statistical analysis showed a statistically significant relationship between ankle/foot pain and load on IV‐V of the metatarsal head in the left foot (*P* = .010); the relationship for the right foot turned out to be statistically insignificant (*P* = .082). Ankle/foot pain also correlated with the load on the medial arch of the left foot (*P* = .011). This relationship was statistically insignificant for the lateral arch (*P* = .084).

Statistical analysis showed statistically significant positive correlations between the BMI coefficient of the study group and the load on the medial arch of the left foot (*P* = .260) and right foot (*P* = .000) as well as the load on the lateral arch of the left foot (*P* = .019) and the right foot (*P* = .340). This may indicate reduced efficiency of the foot arches associated with excessive body weight.

There was also a statistically significant correlation between work experience and neck pain (*P* = .036); older nurses complained about neck pain more often. Correlations between the number of hours spent standing, and neck complaints (*P* = .076) were close to the statistical significance threshold. It is worrying that the pain is already present in young nurses who are just starting their professional careers.

## 4 DISCUSSION

Apart from the specific nature of the nurse's work and the overloading to which they are exposed in the course of their professional activities and duties, there are many other causes of spinal pain. Dr McGill, professor of spinal biomechanics at the University of Waterloo in Canada, has been running a laboratory to evaluate the most common lumbar spinal dysfunctions. He is precursor of the prevention of spinal disorders, rehabilitation, and sports training, as well as gives indications for prophylaxis, including systematic exercises.^27^

An additional factor affecting the movement system is significantly longer working time, often amounting to 12 hours a day. Not without significance are also individual features, shoes used, and genetic factors, level of physical activity, concomitant diseases and past injuries.^28^, ^29^ The prevention of overloading of musculoskeletal structures should cover not only the area of behavior at work but also daily physical activity and the way of spending free time.^30^ The way of loading and setting of the foot is often the result of biomechanical relationships concerning other parts of the movement system. Structural changes in the spine may be the cause of asymmetry of foot loads, weakening of their muscle, ankle stabilization and gait asymmetry.^31^

Annette Verpillot from Montreal in Canada, founder of Posturepro posturology clinic, is a world‐famous specialist of posturology, creator of the concept, neo‐science, pointing to important elements that change the image of the posture. According to her theory, the body receives sensory information from the environment mainly through the feet, eyes, occlusion (setting the joint surfaces of the temporomandibular joint) and skin. Such perception and analysis of changes in the image of posture indicates the great importance of feet and their daily supplies (ie the footwear used).^32^

A review of available Polish literature indicates the need for further research in the area of foot load distribution among active personnel. Functional diagnostics should be based on the results of research with the use of the latest technologies, which enable more and more accurate diagnostics and analysis of gait and posture control of the human body, the employee.

The work of a nurse is an extremely physically and mentally demanding activity. To maintain the health and safety of nursing staff, knowledge and application of ergonomic principles at the workplace is essential. The research by Juraszek et al^33^ on the impact of work on the incidence of spinal pain in nurses shows that as much as 54% declare that they are more familiar with ergonomics at work, 26% of the respondents state that they are familiar with these principles, 13% of the respondents gave the answer "rather no" and 6% "definitely not". Only 8% of respondents declare that they always apply the principles of ergonomics, 47% often, 38% rarely, 7% never. These data are confirmed by our study. The differences may result from too small a group of respondents, but there is a tendency to neglect the knowledge and application of the principles of work ergonomics among nurses.

Moreover, it should be emphasised that our study showed that 68% (n = 25) of respondents declare that they know the principles of ergonomics. However, only 14% (n = 5) admitted that they always apply ergonomic principles in the course of their work. This dependence shows that nurses do not pay attention to the principles of ergonomics at the workplace, despite their knowledge of the subject. This is a worrying piece of information and may indicate that the consequences of non‐compliance with these principles or the lack of time and urgency at work are being underestimated.

An extremely important and worrying issue is the negligent attitude of nursing staff towards compliance with the basic principles of ergonomics in the context of workplace safety should not take place. Specific educational and training programmes are needed, aimed not only at knowledge of the principles of ergonomics but also at instructions on how to apply them effectively in everyday practice. It is important to inform about the health consequences and dysfunction of the movement system in the form of overload and pain syndromes, but this also involves disability due to long‐term neurological consequences of spinal disorders and coexisted nervous tissue irritability. It should be pointed out once again, that the pain syndromes are already present in young nurses who are just starting their professional careers.

It seems worrying that pain is reported regardless of age. Even young nurses who were just entering the labor market indicated a number of these ailments. In the question about how to deal with pain 38% (n = 14) of respondents indicated taking analgesics, only 27% (n = 10) of respondents use physiotherapist's advice and the other as much from biological regeneration. Interestingly and worth emphasizing, none of the respondents indicated a visit to a doctor as a way to deal with pain.

Studies conducted by Maciuk et al^34^ concerning self‐assessment of the occurrence of spinal pain syndromes in professionally active nurses show that as many as 82% of the nurses surveyed felt pain in the lower spine and 53% of the examined cervical pain in the spine. In similar studies conducted by Juraszek et al^33^ as many as 92% of the nurses surveyed declared the feeling of spinal pain and indicated professional activity as the cause of such ailments. The results of our own research are analogic to the quoted data. In the group of nurses, 62% felt pain in the neck area, 57% felt pain in the upper back area, and 73% felt pain in the lower back area. Our study demonstrated that none of the respondents indicated a visit to a doctor as a way to deal with pain. This may be due to limited confidence in doctors in terms of effective and long‐term pain management, the use of rather interim medications for pain relief. This may also be due to the use of analgesics on their own, without the need for a prescription for stronger analgesics and anti‐inflammatory drugs.

The correct matching of everyday footwear is considered essential as unfitting footwear is a common cause of foot pathologies. It has been suggested that incorrectly fitted footwear is a major contributor to the development of structural foot disorders such as hallux valgus and smaller finger deformations. Globally, it also affects the abnormal position of the lower limbs, which affects the angle of inclination of the pelvis and generates disorders overloading the lumbar spine.^35^ Footwear is the only connection between the body and the ground in a standing position thus changes in footwear may affect the forces acting through the body, posture and movement, especially for those performing standing work.^36^, ^37^ Our study documented that only 43% (n = 16) use a proper, profiled medical footwear, ensuring an optimal conditions for the proper functioning of the foot during standing and walking. This problem should finally be noticed and effectively highlighted because wearing comfortable nursing shoes is essential to reduce lower limb discomfort for clinical nurses and to reduce the incidence of musculoskeletal disorders.

Due to the low availability of studies on the distribution of foot load during walking among active nurses, studies conducted on different groups of patients will be quoted for further consideration. Wafai et al^26^ studies on the symmetry of foot load during pathological and normal gait showed significant differences between the distributions of foot load. The level of asymmetry of foot load in pathological walking was much higher than in the control group. The areas of the greatest load asymmetries were the III and V metatarsal bone and the lateral part of the heel. Foot pathologies, manifested by pain and improper positioning and functioning of the foot influence the asymmetry of the transmitted load. The results of the observations showed a relationship between ankle/foot pain and excessive asymmetric load on specific areas of the feet.

Our study showed reduced pressure in the area of the first head of metatarsal bones; increased pressure occurred in the area of the second and third head of metatarsal bones. It seems interesting that there is a certain regularity in the distribution of pressure on feet between a group of people with toenail hallucinations and a group of nurses, however, due to a small group of patients and a different examination procedure, the conclusions should not be consolidated.

Excessive body weight is a factor that predisposes to the formation of degenerative changes in the joints and the formation of the flatfoot and many other deformed changes in the foot. A study of standing load distribution of feet conducted by Lorkowski et al^38^ among women with obesity and inflammation of the sole tendon showed a significant increase in the load in the areas of the metatarsal bone, lateral and medial arch of the foot and the anterior part of the plantar foot. Our own research conducted during the gait showed a statistically significant relation between the BMI index and the occurrence of increased pressure in the lateral and medial arch of the foot. The BMI coefficient correlated positively also with the following parameters: average foot surface, an average of maximum foot loads and an average of average foot loads for both left and right foot.

An interesting study was presented by Baumfeld et al,^39^ in which the nurses working in the hospital were subjected to static baropodometric tests. The whole procedure consisted of four measurements: in the first part of the foot load test was performed before work and after a 12‐hour shift at work, in the second part of the test was performed before and 10 minutes after the exercises are stretching the posterior group of lower limb muscles. The analyzed data did not show statistically significant differences between the load on the forefoot and the backfoot in both feet. Respondents concluded that in the case of fully fit and healthy people who have not experienced any injuries to the musculoskeletal system, neither hard physical work nor a series of stretching exercises could change the distribution of forces on the feet. The data are not confirmed by our own study.

The research by Kenny et al^40^ on foot load distribution carried out on research workers consisted of two procedures: a static and a dynamic baropodometric test. The relationship between the pressure on the sole side of the feet and the shape of the foot, the arches of the foot and individual deviations from the normal posture of the body were demonstrated. Shortened calf muscles translate into overload on the backfoot and disturbances in the balance of biokinetic chains. The BGA test, which combines static and dynamic tests, provides an opportunity for in‐depth analysis of the foot and the entire movement system.

A study by Caravaggi et al^41^ on the relationship between the normal range of motion of the foot joints and the correct pressure on the sole side of the foot during gait showed a negative correlation between the pressure around the foot and forefoot and the range of motion in the ankle‐metatarsal joint. Less mobility of this joint affects the increased pressure under the front and rear part of the foot. The presented data are not confirmed by our own research.

The results of our research indicate the need for a holistic approach in the process of monitoring the health behavior of nurses. The study shows that the occurrence of pain in the cervical region of the spine affects the abnormal load on the feet during gait. Thus, the observations proposed by Verpillot,^42^ pointing to a series of correlations of the work of individual posture control systems, relating to the reaction of the body to a wrong signal that the brain receives and analyzes, is justified.

Training and implementation of an appropriate model of ergonomics of work, based on the latest reports, should become a binding standard to prevent inappropriate behavior in the course of professional activities.

### 4.1 Study limitations

The study should be extended to a larger group of active nurses including comparisons with the control group to obtain more statistically valid results. Moreover, future studies should test temporo‐spatial parameters of dynamic BGA assessment during gait such as gait cadence [steps/min], gait speed [m/s], and gait cycle for subsequent phases [%]. Also, it is well known that differences between dominant and non‐dominant sides in static dynamic BGA assessments exists,^43^ thus the influence of the dominance of the lower limb on BGA results should be taken into consideration. Another issue is to compare the obtained results to male nurses. It was shown that contact area in males is larger in all regions of the foot compared with females, however, there are no between gender differences in peak pressure, contact time, pressure‐time integral and instant of peak pressure.^44^ Nevertheless, the difference between genders should be studied with BGA in relation to low back pain and occupational conditions. And the last, but not least other objective measurement tools, useful in assessing the impact of low back pain for postural disturbances of the body can be used, such as stabilographic platform.^45^, ^46^

## 5 CONCLUSION

A group of nurses tested put an abnormal strain on the feet. There are noticeable disproportions between the loads on the three main support points of the foot. The load on I metatarsal bone head was laterally shifted to the area of II and III metatarsal bone head.

## DISCLOSURE

*Approval of the research protocol*: The study was approved by the independent Bioethics Committee at the Wroclaw Medical University in Poland (no. KB–148/2018). The study was conducted according to the Declaration of Helsinki and Good Clinical Practice guidelines. *Informed consent*: The study was completely anonymous, and each of the nurses surveyed gave voluntary and written informed consent to participate in the study. *Registry and the registration no. of the study/trial*: N/A. *Animal studies*: N/A. *Conflict of interest*: Authors declare no conflict of interests for this article.

## AUTHOR CONTRIBUTIONS

Study design: AK and NG Data collection: NG Data analysis: AK and MK Manuscript writing: AK and NG Revisions for important intellectual content: AK and MPB.
